# Neuroendocrine Tumors: An Analysis of Prevalence, Incidence, and Survival in a Hospital-Based Study in Ecuador

**DOI:** 10.3390/healthcare10081569

**Published:** 2022-08-18

**Authors:** Karime Montes Escobar, Jose Luis Vicente-Villardon, Rosa Elisa Villacís Gonzalez, Paul Humberto Castillo Cordova, Johanna Mabel Sánchez Rodríguez, Melina De la Cruz-Velez, Aline Siteneski

**Affiliations:** 1 Department of Mathematics and Statistics, Institute of Basic Sciences, Universidad Técnica de Manabí, Portoviejo 130105, Ecuador; 2 Statistics Department, University of Salamanca, 37007 Salamanca, Spain; 3 Teaching and Research, SOLCA, El Oro, Machala 070206, Ecuador; 4 Teaching and Research, SOLCA, Loja 110150, Ecuador; 5 Facultad de Medicina, Universidad Laica Eloi Alfaro de Manabí, Manta 130203, Ecuador; 6 Facultad de Ciencias de la Salud, Universidad Estatal del Sur de Manabi, Jipijapa 130650, Ecuador; 7 Faculty of Health Sciences, Medicine Career, Universidad Técnica de Manabí, Portoviejo 130105, Ecuador; 8 Research Institute, Universidad Técnica de Manabí, Portoviejo 130105, Ecuador

**Keywords:** incidence, neuroendocrine tumor, population, prevalence, survival, HJ-Biplot

## Abstract

Neuroendocrine tumors (NETs) represent a heterogeneous malignancy group of neoplasms, with a limited amount of data from Latin America. Thus, this observational study aimed to provide data about the prevalence, incidence, and survival rates for NET in Ecuadorian hospitals. The study was conducted using data from the Society for the Fight Against Cancer (SOLCA). We evaluated patients with NETs (2000–2020) using the HJ-Biplot method and Cox proportional hazards. Annual age-adjusted incidence and limited-duration prevalence in multivariable analyses as well as hazard ratios (HRs) for mortality and survival were obtained. In the years 2000–2020, the age-adjusted incidence rate increased by 9-fold in the stomach and by 7-fold in the breast. The incidence rates were 1.38 per 100,000 persons in the lung and at 1.79 per 100,000 persons in gastroenteropancreatic sites (rectum, stomach, and pancreas). The prevalence increased from 0.0027% in 2000 to 0.0736% in 2019 and 0.0245% in 2020. Overall survival was worse for metastatic NETs (HR, 4.061; 95% CI, 1.932–8.540; *p* < 0.001) and advanced local NETs (HR, 2.348; 95% CI, 1.007–5.475 *p* < 0.048) than for localized NETs. In conclusion, the NET incidence increased in the last 20 years and survival decreased over time, especially for metastatic tumors in the pancreas and the nostril.

## 1. Introduction

Neuroendocrine tumors (NETs) are relatively rare tumors that are considered potentially malignant, with primary tumors being derived from neuroendocrine cells; they are found in several organs throughout the body [1]. From a distance, neuroendocrine cells exert a regulatory function on target organs that have an endocrine function; besides their paracrine function at the local organ level, the role is often of unknown specific significance [2]. Frequently, NETs originate in the gastrointestinal tract, lungs, pancreas, and thyroid [3,4,5,6], and patients above the age of 65 present a higher incidence [7]. NETs are a heterogeneous group of neuroendocrine neoplasms that differ from neuroendocrine carcinomas, and the prognosis significantly varies among subgroups [8]. Commonly, NET recognition comes from pathologic classification, in grades based on their morphologic features [8,9,10]; the low grades signify less risk of distant metastases, and the high grades are more aggressive with a poor prognosis [11].

Recently, the incidence and prevalence of neuroendocrine tumors (NETs) have grown, owing to, among other aspects, the increased diagnosis rates of currently available diagnostic techniques such as computed tomography and endoscopy [12,13] for early-stage tumors [14]. Globally, the highest rates of increase have occurred in countries such as the United States, Canada, and Norway [15]. Over the last four decades, the incidence of NETs has increased more than sixfold in the United States alone, with a tendency toward localized tumors rather than metastatic tumors. Approximately 170,000 people were diagnosed with NETs in the United States in 2012 [16,17]. Gastroenteropancreatic sites represent the most common NET subtype, comprising 55–70% of all NETs [14].

Particularly noteworthy is the fact that the evidence of NET prevalence and incidence in Latin American countries is very scarce [18,19]. In Argentina, an observational study evaluated a hospital database and showed that among the 532 patients diagnosed with NETs, 86.6% were gastroenteropancreatic NETs and 13.4% were bronchial NETs, with a median age of 53.2 months and an overall survival time for gastroenteropancreatic NET patients of 65.1 months [19]. A similar study was conducted in Chile, and the report evaluated 166 NET patients; they observed the prevalence of NETs among males, with an average age of 53 years and an overall survival time of 110 months. The NET primary tumor sites were found in the gastroenteropancreatic system and the small bowel [20]. Recently, two cohorts were evaluated in Panamá between 2016 and 2019, with NET occurrence at a mean age of 60 years, with a diagnosis grade of G1, and with the liver as the organ most affected [21]. Finally, a Brazilian registry of 1000 patients was evaluated over 33.7 months; the survival rate in the study was 29.3%, and the prevalence was 71.6% for thoracic NETs and 20.2% for gastroenteropancreatic tumors [18].

The heterogeneity of NETs has resulted in many different rates of prevalence and incidence, determined by the predominant site of the tumor, the vulnerable population, ethnic differences, and lifestyle. These varied aspects demonstrate the need for national and regional databases [18,19,20,22]. Ecuador is one of South America’s smallest nations, lying on the west coast, with a total size of 283,561 km^2^ (Central Intelligence Agency, The World Factbook, South America, 2018). It is geographically divided into four large sections: the Amazon, the Highlands, the Coast, and the Galapagos Islands. It is politically divided into 24 provinces with 224 cantons (Instituto Nacional de Estadística y Censos. Población y Demografía, 2018). In Ecuador, the Cancer Fighting Society (SOLCA) has maintained registry data of cancer prevalence and incidence since 1984 (Ministerio de Salud Pública). There are six hospitals distributed throughout the main provinces that care for approximately 31% of Ecuador’s oncological cases [23]. Evidence of NET prevalence or incidence in Ecuador is scarce; for example, the global surveillance of cancer survival for the period of 1995–2009 that analyzed 67 countries, including Ecuador, did not report NET cases, probably because of the low incidence or prevalence rates [24].

In this context, the aim of our study was to evaluate the prevalence, incidence, and survival rate of NETs among Ecuadorian patients. In addition, we investigated the predominant organs affected by NETs and the impact of the histological differentiation proposed by WHO in 2010, the classifications into grades G1/G2/G3 (proliferative index Ki-67), and the primary sites of the NETs. For this purpose, 20 years’ worth of data of registered patients at SOLCA hospitals were analyzed with classical and distinct statistical Cox proportional hazards and the HJ-Biplot method.

## 2. Materials and Methods

The retrospective cohort study was accomplished with a clinical story assay of NET patients. The Cancer Fighting Society (SOLCA) databases of the Manabí, Cuenca, Loja, and El Oro provinces were analyzed. All NET patients who had been diagnosed and attended to at the Oncology Service of SOLCA centers between January 2000 and December 2020 were included. Patients who did not have a complete clinical story, abandoned treatment, or did not follow treatment in the hospital were excluded. Here, we assessed age, sex, population, stage and differentiation grade of tumor, year of diagnosis, and survival of the NET patients (measured in months or until death). The clinical and pathological characteristics of the study population are detailed in Table 1.

### 2.1. NET Classification

The NETs were analyzed according to the WHO (2010) histological grades: Grade 1 (G1, Ki-67 index <2% and mitotic count <2/10 high-power fields (HPFs)), Grade 2 (G2, Ki-67 index 3–20% and/or mitotic count 2–20/10 HPF), and Grade 3 (G3, Ki-67 index 3–20% and/or mitotic count 2–20/10 HPF), and G3 (G3 neuroendocrine carcinoma were not differentiated), notwithstanding tumor differentiation [8,25]. In addition, the tumor stages of NET patients were assayed as local, advanced local, and metastatic. The local tumor was within the limits of the organ of origin; the advanced local tumor expanded beyond the organ of origin into adjacent tissues or organs or infiltrated regional lymph nodes; finally, the metastatic tumor was one that extended to remote sites outside the organ of origin.

### 2.2. Statistical Analysis

The examination of survival at 20 years according to site was performed using the Kaplan–Meier method and the log-rank test. In addition, we evaluated the data from the Sociedad de Lucha Contra el Cancer (SOLCA) hospital registry to extract the median overall survival (OS) by site, stage, and grade. To evaluate the most recent trends in survival, we conducted multivariable survival analyses. Five-year overall survival (OS) time and the Cox proportional hazards model were used in the multivariable analysis, with censoring applied at 5 years. Covariates for this analysis included factors known to influence the prognosis of NETs, including grade, age, stage, site, and time interval from diagnosis. The overall model was significant at *p* <  0.001.

Incidence (including annual percentage change) and limited-duration prevalence rates (20 years) were calculated using the annual percentage change by fitting a least-squares regression to the rates, using the calendar year as a regression variable. Age-adjusted incidence rates were computed using weighted proportions of the corresponding age groups in the Ecuadorian standard population for each province: El Oro (715,751), Manabí (1,562,079), Lora (521,184), and Azuay (881,394), with a total of 3,670,408 (Instituto Nacional de Estadistica y Censos, 2020).

### 2.3. HJ-Biplot

**Figure 1:**
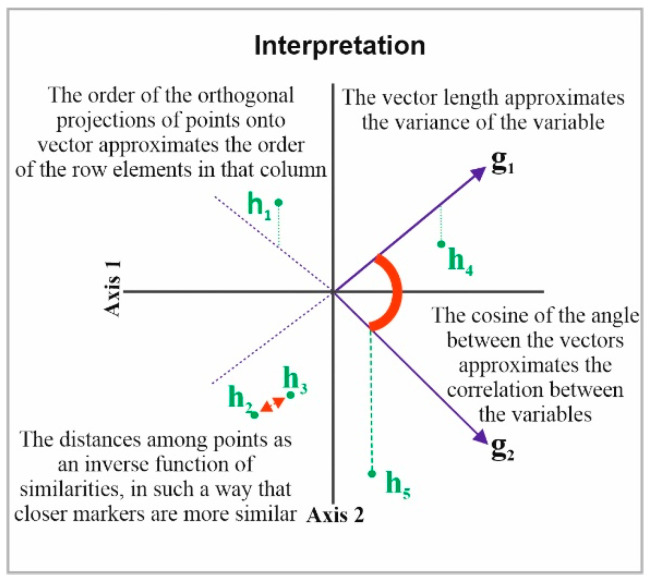
Description the HJ-Biplot method.

Next, the HJ-Biplot [26] was used, which is an extension of the classic biplot introduced by Gabriel [27] and is an exploratory data analysis method that searches for hidden patterns in the data matrix. The HJ-Biplot has the advantage of simultaneously being a representation and achieving an optimal rendering quality for both rows and columns, with both represented in the same reference system (Figure 1). The data matrix then graphically shows the information contained in the rows (sample location) and columns (grade and months). The Biplot is a multivariate statistical technique that was performed in order to allow for a more detailed data analysis, highlighting the links between parts (sample location) and the gender and age of the individuals.

Statistical analyses were performed using IBM SPSS version 25.0 (IBM, Armonk, NY, USA), R software version 3.3.1 (http://www.r-project.org; accessed on 30 June 2022), and the MultBiplot freely available software [28]. Comparative differences were considered significant at *p* < 0.01.

## 3. Results

Between 2000 and 2020, 281 admissions corresponding to patients affected by NETs were registered in the database of SOLCA clinics in four provinces of Ecuador. We observed that the most common NETs were those of the lung (18.1%), stomach (12.1%), breast (9.3%), nasal cavity (7.1%), and rectum (6.4%). The database permitted the analysis of tumor grade according to the WHO classification; most of the cases were at the advanced stage G3 (56%), followed by G2 (25%), and G1 (20%). The primary tumor sites that most frequently presented with stage III disease were the lung (21%), stomach (8.9%), pancreas (8.9%), nostril (7%), unknown (7%), and finally, the breast (6.4%) (Table 1).

### 3.1. Annual Incidence

**Figure 2:**
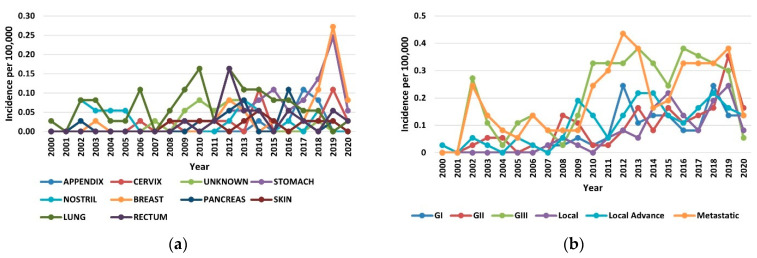
Incidence trends of neuroendocrine tumors (NETs) from 2000 to 2020: (**a**) Annual age-adjusted incidence of NETs by site; (**b**) Annual age-adjusted incidence of NETs by stage and grade.

The increase in the incidence of NETs in the period of 2000–2020 occurred across all organs, stages, and grades. In the year 2019, the increases in incidence for various sites ranged from 8-fold in the breast to 5-fold in the stomach (Figure 2a). Among the stage groups, the incidence increased the most for metastatic NETs, from 0.24 per 100,000 persons in 2000 to 0.38 per 100,000 persons in 2020 (*p* < 0.001) (Figure 2b). The highest incidences were 1.38 per 100,000 persons for the lung, 1.79 per 100,000 persons for gastroenteropancreatic sites (including 0.92 per 100,000 persons for the stomach, 0.49 per 100,000 persons for the rectum, and 0.38 per 100,000 persons for the pancreas), and 0.40 per 100,000 persons for NETs with an unknown primary site of origin.

### 3.2. Prevalence

**Figure 3:**
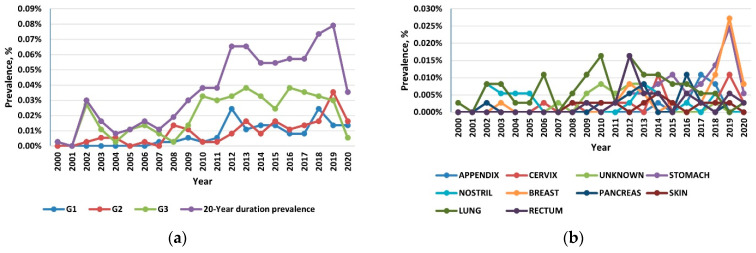
(**a**) The 20-year limited-duration prevalence of all neuroendocrine tumors (NETs), according to grade; (**b**) The 20-year limited-duration prevalence of neuroendocrine tumors by site.

Reflecting the rising incidence and indolent nature of NETs, the 20-year limited-duration prevalence increased substantially, from 0.0027% in 2000 to 0.0736% in 2019 and 0.0245% in 2020 (*p* < 0.001) (Figure 3a). Among the grade groups, the prevalence increased the most for the G3 NETs, and among the sites, prevalence was the highest in the lung, followed by the stomach and the pancreas (Figure 3b).

### 3.3. Survival

**Figure 4:**
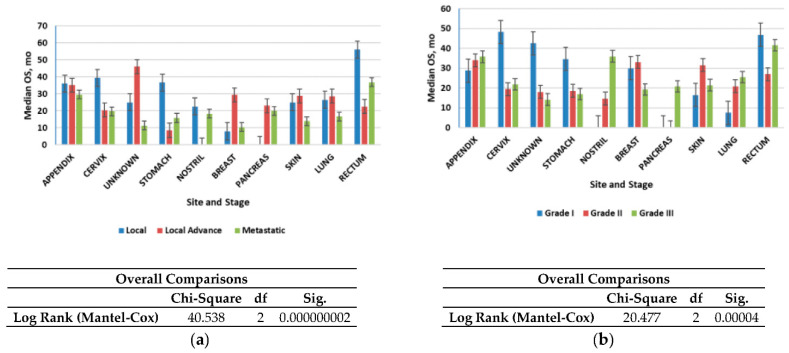
Median overall survival (OS) of neuroendocrine tumors (NETs): (**a**) Median OS of all patients included in the study according to stage; (**b**) Median OS of all patients included in the study according to grade. Error bars indicate 95% CI.

Figure 4a shows that the median OS time for all patients was 2.4 years (28.8 months); localized NETs had a better median OS at 2.30 years (26.6 months) as compared with advanced local NETs at 2.14 years (25.7 months) and metastatic NETs at 1.60 years (19.2 months) (*p* < 0.001). Of those with known grades, G1 NETs had the highest median OS (2.12 years) (25.4 months) among the grade groups, G2 NETs had a worse OS at 2.07 years (24.9 months), while G3 NETs had the worst OS (1.8 years). NETs in the rectum (3.6 years) and appendix (2.7 years) had the best median OS among the site groups, while NETs in the pancreas (1.19 years) and nostril (1.14 years) had the worst median OS. All these differences in survival were significant (log-rank *p* < 0.001).

We then evaluated the survival patterns according to site and stage (Figure 4b). In the localized NETs, the median OS ranged from 3 years in the small intestine to more than 4.6 years in the appendix. In advanced local NETs, the median OS ranged from 2.9 years for NETs in the appendix to more than 30 years in the unknown primary site. For distant NETs, those in the rectum had the best median OS (3 years); NETs in the appendix (2.4 years) and pancreas (1.6 months) had the worst median OS. All of these differences in OS were significant (log-rank *p* < 0.001) (Figure 4b).

### 3.4. Multivariable Analysis of OS

**Figure 5:**
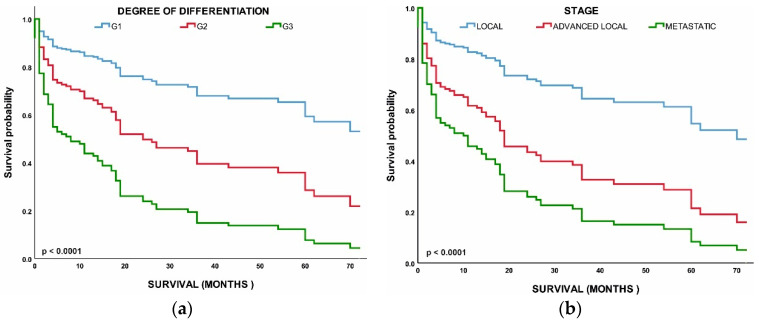
(**a**) Survival of patients by tumor grade: grade 1 (G1), grade 2 (G2), grade 3 (G3); (**b**) Survival of patients by tumor stage: local, advanced local, and metastatic.

Next, we performed a multivariable analysis with hazard ratios (HRs) calculated for 5-year mortality hazard rates (Table 2). We found that patients with G2 (HR, 2.407; 95% CI, 0.941–6.154; *p* < 0.037) and G3 (HR, 5.003; 95% CI, 2.221–11.273; *p* < 0.001) NETs had worse OS than those with G1 NETs. Age, stage, and grade were all found to have a significant correlation with survival. Overall survival was worse in metastatic NETs (HR, 4.061; 95% CI, 1.932–8.540; *p* < 0.048) and advanced local NETs (HR, 2.348; 95% CI, 1.007–5.475; *p* < 0.001) than in the localized NETs. All of the above comparisons were significant at *p* < 0.001 (Figure 5).

### 3.5. HJ-Biplot

**Figure 6:**
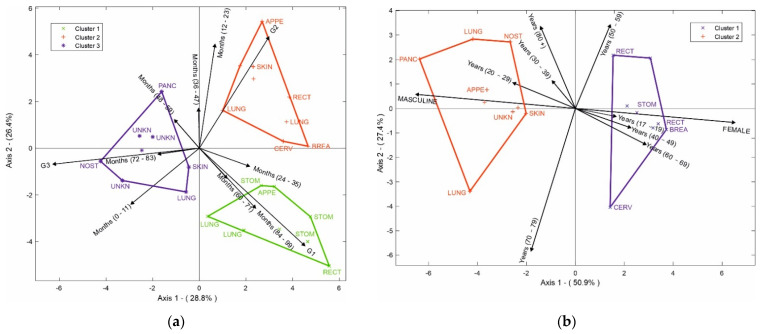
(**a**) HJ-Biplot relating the degree of differentiation and months of survival, according to tumor location; (**b**) HJ-Biplot relating gender and age according to tumor location.

In addition, the HJ-Biplot method was used (Figure 6a) to determine the relationship among variables, considering the 10 most prevalent NETs. The degree of differentiation, months of survival, and the location of the tumor were evaluated. In the degree of differentiation for G1, the highest survival rates occurred between 84 and 99 months; tumors in the stomach and appendix had a high correlation with 24–35 months of survival, while tumors in the rectum and lung had an even higher survival rate at 84–99 months. When we evaluated the degree of differentiation for G2, the highest survival was observed between 12 and 23 months in NETs in the appendix, lung, breast, and cervix. Finally, we evaluated the degree of differentiation for G3; the skin had the worst prognosis, with a survival range between 0 and 11 months, a survival range between 48 and 59 months for the pancreas, and between 72 and 83 months for nasal cavity tumors together with NETs of unknown origin.

Figure 6b shows age, gender, and location of the NETs where two groups were observed. The first group represents the female gender, which had a significant correlation with the cervix, breast, stomach, and rectum tumors. The second group was the male gender, which was correlated with tumors of the pancreas, unknown site, skin, lung, nostril, and appendix. It was possible to determine that the rectum NETs for the female gender were related to ages between 50 and 59 years, while the stomach, breast, and cervix NETs had a higher connection with women between 60 and 69 years. For men, the nasal fossa tumor appeared in individuals over 80 years old, NETs of the pancreas, appendix, skin, and unknown sites had a greater connection with young people between 20 and 29 years old, and lung tumors appeared between the ages of 70 and 79 years.

## 4. Discussion

In this study of NETs using data from an Ecuadorian center, we assessed 20 years’ worth of data and found that the age-adjusted annual incidence increased for metastatic NETs from 0.24 per 100,000 persons in 2000 to 0.38 per 100,000 persons in 2020. Prevalence rates grew from 0.0027% in 2000 to 0.0736% in 2019 and 0.0245% in 2020. A decrease in NET prevalence in 2020 as compared with 2019 may have been a consequence of undercounting due to the COVID-19 pandemic. Unfortunately, our data showed that advanced-grade NETs showed the highest prevalence, with main sites in the lung, stomach, and pancreas. Additionally, our NET survival rate showed a low median at 2.4 years for all patients; the rates were better for localized NETs and the worst for metastatic NETs. These data on patients’ survival may reflect the public and private structure of the health system in Latin America, where public policies encouraging health care and access to cancer treatment are still scarce [29].

This retrospective cohort study examined the general incidence increases—9-fold for the stomach and 7-fold for the breast—between 2000 and 2020. Prior studies carried out worldwide have also reported that the NET incidence increased 6.4-fold in 39 years; their data reported an increase from 1.09 per 100,000 in 1973 to 6.98 per 100,000 in 2012 in the United States [14]. According to a Kentucky study, a 20-year database showed the rise from 3.1 in 1995 to 10.3 in 2015 per 100,000 cases of NET patients [22]. In fact, differences in population rates may result from a combination of environmental factors and biological differences owing to differing national demographics [30,31,32]. Ecuador is positioned on the equatorial landline, has territory in both hemispheres, and is traversed from north to south by the Andes Mountains. It has approximately 17.511.000 habitants, with a predominant mestizo (descendants of Indigenous Americans and Spaniards) population. In Ecuador, the Quito SOLCA database revealed that between 2011 and 2015, NETs represented 1% of total cancer cases per 100,000 inhabitants, and the Guayaquil SOLCA data reported no specific NETs in their published reports [33]. Our study considered the SOLCA database of NETs patients from four large centers of the country, which were assessed for a period of 20 years. In the Ecuadorian population, the most common site of origin for NETs was the lung, followed by the gastroenteropancreatic system.

Previous data demonstrated that mostly gastroenteropancreatic NET tumor sites surged, as reported in the EUA database, from 1.09 per 100,000 in 1973 to 6.98 per 100,000 in 2012 [14]. It is important to highlight that the growth in incidence data can be attributed to modern imaging techniques that favor appropriate diagnoses and an improved recognition of neuroendocrine histology [14,15]. In fact, gastroenteropancreatic NETs represented the most common site of origin when standardizing our data. Worth noting is the fact that gastroenteropancreatic NETs represented the second most prevalent digestive cancer [5]. Pancreatic NETs originating in Langerhans islets or alternative origins in precursors in the ductal epithelium [34,35,36] because of malignance represented the most widely studied NETs between 1981 and 2020 [37]. Our data also suggested poor survival prognosis among pancreatic NET patients, at only 1.19 years after diagnosis. On the other hand, the NET site of origin prone to the earliest diagnosis was the stomach.

A relevant consideration of our incidence analysis was the high rate of lung NETs, with 1.38 per 100,000 habitants. In this study, we observed that the lungs represented the most prevalent site for NETs. In the lungs, the NETs originate in the amine precursor uptake and decarboxylation of neuroendocrine cells from Kulchitsky cells [38] and account for around 20% of all lung malignancies [39]. In addition, pulmonary tumor morphologies are different when compared to other NETs, with the presence of rosette-like structures, organoid nesting, and peripheral palisading patterns [39]. A recent retrospective analysis showed the lung as the second most likely NET site in the USA, with the highest rates from 2000 to 2012 at 1.49 per 100,000 [14]. An increase of up to 6% per year was observed for pulmonary NETs in the USA [40]. Another study performed in the USA showed a 7% yearly rise in the incidence of lung NETs between 2004 and 2014 [41]. In particular, the primary tumor sites that most frequently presented with severe stage (G3) disease in our data were the lungs. The overall prognosis of our data was not favorable for NET patients, with over half of tumor diagnoses being in the advanced stage.

NETs are malignant heterogeneous tumors originating from cells of the diffuse neuroendocrine system that can show both nerve and endocrine cell features and are found in many organs in the body [42]. Nevertheless, several risk factors are associated with tumor incidence; for example, a family history of neuroendocrine tumors of endocrine neoplasia [43,44]. More frequently found are clinical symptoms associated with NETs such as cough, dyspnea, weight loss, diarrhea, constipation, abdominal pain, dyspepsia, hypertensive crisis, distended abdomen, and intestinal obstruction [45]. NETs are often identified based on phenotypical and morphological features consistent with the WHO classification [39], which is commonly revealed by employing immunostaining with neuroendocrine markers and Ki-67. In the current WHO categorization, synaptophysin, chromogranin A, and CD56 are recommended as neuroendocrine indicators, with synaptophysin and chromogranin A being recommended as first-line options [39]. NETs have distinct clinical and biological properties depending on where they originate; for instance, those originating from enterochromaffin cells characterized as G1 NETs are part of the diffuse neuroendocrine system and may present a prevalence of 39.2% for GI NETs of the rectum and 27.8% for the small intestine [46]. In this context, our results suggest that 56% of NETs are diagnosed in the G3 stage, most frequently in the lung, followed by the stomach and pancreas. Nowadays, predictive and prognostic markers have been extensively researched in order to determine a better clinical management strategy for NETs [47].

General radiology methods such as more sensitive computed tomography and magnetic resonance imaging, in addition to the best therapies for gastrointestinal NETs, were previously reported as responsible for the increased survival indices [12,13]. Unfortunately, in our data, the low survival rate found among NET patients studied for 2.4 years could represent the advanced stage of tumor diagnosis. However, our data represent a low median at 2.12 years (25.4 months) for G1 NETs and 1.8 years (21.6 months) for G3 NETs as compared with other population studies that have survival rates of >30 years for regional diseases and 12 months for patients with distant metastases [14]. Evidently, the survival rate was reduced in the more advanced stage of the disease and diagnosis at a high grade; indeed, we observed small survival rates of 1.19 years for the pancreas and 1.14 years for the nostril.

Our study also has some limitations. First, the characteristics found in our sample may not be representative of the performance in other centers of the country. However, in Ecuador, the Instituto Nacional de Estadistica y Censos (INEC) collects, analyzes, and reports statistical information on health, including cancer deaths, but it does not report on NET specifically. Second, INEC uses the International Classification of Diseases, tenth revision (ICD-10), to report the causes of death. This classification provides codes for causes of cancer death, but not for NET specifically. There is a possibility that the ICD-10 includes NET in a wide range of endocrine, nutritional, and metabolic diseases, which makes the use of such database in our evaluation difficult. Third, we collected the SOLCA data from four important regions of Ecuador, and we acknowledge that this may not show the real scenario of NET patients. SOLCA data from Quito and Guayaquil were missing because representatives from these sites refused to participate in our research. For these reasons, our results on prevalence, incidence, and survival may underestimate the real number of NET patients in Ecuador. Our data represent an upper ceiling to the high proportion of G3; in fact, this may have contributed to the median survival time of 2.4 years, which is significantly worse than what is known for the classical distribution of NET G1/G2. Finally, the current WHO (2019) classification defines NETs as well-differentiated and poorly differentiated. Well-differentiated NETs are based on the mitotic rate and/or Ki-67 labeling index and the grades of G1/low, G2/intermediate, and G3/high. Poorly differentiated ones are G3 NETs in neuroendocrine carcinomas of small or large cell carcinoma [48]. The WHO 2019 classification did not use in our database evaluation between 2010 and 2020. New studies are necessary to provide the NET current WHO classification in the Ecuadorian population.

The findings of this study have important implications for NET patients, for communities in the country, healthcare systems, and future research. Based on the results of this study, the NETs are defined as a serious type of cancer with low survival times and with its prevalence increased in the last two decades. Especially in low-income and mid-income countries, understanding the prevalence of NETs is crucial in changing public policies and setting priorities in order to improve patient care. Therefore, the estimates of survival time in this study and the identification of the characteristics of patients most affected by NETs as well as the more common sites of the disease may help to guide physicians in making early diagnoses among Ecuadorian populations. Although relatively rare, our results highlight the severity of NETs, and the data presented may contribute to changes in public policies in the country. This is the first study on NETs in Ecuador, and the data on incidence and prevalence must be considered as preliminary. More research is necessary at a national scale to ensure that the whole population is represented.

## 5. Conclusions

The incidence of NETs increased in Ecuadorian hospitals between 2000 and 2020, and we provided primary data on their prevalence. Our data showed that the drastic reduction in diagnosed cases of NETs in 2020 reflected the drop in the number of people seeking health services owing to the worldwide COVID-19 pandemic. It is possible to assert that the statistical HJ-Biplot method we used confirmed our results, and the classical evaluation and proportional hazards model effectively determined the survival rates. Unfortunately, the advanced stages of NETs during the diagnosis of patients in Ecuador contributed to the low survival time observed in our data. The survival was greater for those with localized NETs, while metastatic tumors presented a worse prognosis, particularly in the pancreas and nostril NETs. It is possible that for the Ecuadorian population, standard diagnosis procedures with a focus on the main NET sites found in our study may be implemented.

## Figures and Tables

**Table 1:** Characteristics of study population.

Characteristics	All Patients	%
	*N* = 281	100
**Gender**		
Men	155	55
Women	126	45
**Age (range)**	**Median = 61.6**	
10–19	5	2
20–29	6	2
30–39	18	6
40–49	18	6
50–59	57	20
60–69	56	20
70–79	64	23
80+	57	20
**Localization**		
Lung	51	18.1
Stomach	34	12.1
Breast	26	9.3
Nostril	20	7.1
Rectum	18	6.4
Cervix	16	5.7
Unknown	15	5.3
Pancreas	14	5
Appendix	13	4.6
Skin	11	3.9
Duodenum	9	3.2
Anus	8	2.8
Cecum	8	2.8
Colon	8	2.8
Mediastinum	8	2.8
Lymph Nodes	5	1.8
Liver	5	1.8
Retroperitoneum	3	1.1
Pyloric Antrum	1	0.4
Coledocus	1	0.4
Endometrium	1	0.4
Exocervix	1	0.4
Ileum	1	0.4
Pelvis	1	0.4
Prostate	1	0.4
Soft tissues	1	0.4
Bladder	1	0.4
**WHO 2010**		
G1	56	20
G2	69	25
G3	156	56
**Stage**		
Local	52	19
Advanced Local	78	28
Metastatic	151	54
**Survival**		
Alive	67	23.8
Dead	214	76.2
**SG** (Overall survival in months) Total cases	Median: 28.70	7.5
**SG** (Overall survival in months) 5 years	Median: 21	16

**Table 2:** Cox proportional hazards regression model for overall survival (5 years).

Variable	Estimate	Hazard Ratio	95% CI		*p* Value
Age (years)	0.023	1.023	1.004	1.042	0.015
**WHO 2010**					
G1	-	Ref	Ref	Ref	0.000
G2	0.878	2.407	0.941	6.154	0.037
G3	1.610	5.003	2.221	11.273	0.001
**Stage**					
Local	-	Ref	Ref	Ref	0.001
Advanced Local	0.853	2.348	1.007	5.575	0.048
Metastatic	1.402	4.061	1.932	8.540	0.001

## Data Availability

Due to nature of the data extracted, all patients were previously anonymized. This is considered a low-risk investigation because it is only a review of medical records. Data management was carried out under the Habeas Data law for security of information management. We did not have access to the personal data of the patients.
